# Association Between Student Purchases of Beverages During the School Commute and In-School Consumption of Sugar-Sweetened Beverages, San Francisco Bay Area, 2013

**DOI:** 10.5888/pcd12.150306

**Published:** 2015-12-17

**Authors:** Anna H. Grummon, Ariana Oliva, Karla E. Hampton, Anisha I. Patel

**Affiliations:** Author Affiliations: Ariana Oliva, California Food Policy Advocates, Oakland, California; Karla E. Hampton, Enigami Ventures, Richmond, California; Anisha I. Patel, Department of Pediatrics and the Philip R. Lee Institute for Health Policy Studies, University of California, San Francisco, California.

## Abstract

**Introduction:**

Consumption of sugar-sweetened beverages (SSBs) such as sodas, fruit-flavored drinks, and sports drinks is a major contributor to childhood obesity. One strategy to reduce children’s SSB consumption has been to restrict the sale of SSBs in schools. However, such policies may not sufficiently curb students’ SSB intake, because students can obtain SSBs elsewhere, including from stores located on their school commute. Little is known about students’ purchases of beverages during the school commute or about whether this purchasing behavior is related to in-school SSB consumption. The objective of this study was to describe where students from low-income, ethnically diverse communities obtain the SSBs they drink during school lunchtime and to examine whether students who purchase beverages while traveling to and from school are more likely to drink SSBs during school lunchtime.

**Methods:**

We analyzed survey data from a random sample of low-income, ethnically diverse middle school students (N = 597) who participated in a randomized controlled trial of a water promotion intervention. We used logistic regression analysis to examine the association between students’ purchase of beverages during the school commute and their SSB consumption during school lunchtime.

**Results:**

One-fifth (20.4%) of students drank an SSB during lunch. Approximately 23% of SSBs were obtained during the school commute. Students who reported buying beverages during their school commute (50.1% of all students) were more likely to report drinking SSBs during lunch than students who reported that they do not buy beverages during the school commute (adjusted odds ratio 3.32, 95% confidence interval, 2.19–5.05, *P* < .001).

**Conclusion:**

Students’ purchase of beverages during the school commute was strongly associated with SSB consumption during school lunchtime. Interventions could benefit from focusing on retail environments (eg, encouraging retailers to promote healthy beverages, posting beverage calorie information).

## Introduction

Nearly one-third of US children aged 2 years or older are overweight or obese (1). Consumption of sugar-sweetened beverages (SSBs) such as sodas, fruit-flavored drinks, sport drinks, and other beverages with added sugar (2) contributes to childhood obesity (3,4). Accordingly, there is increasing interest in interventions to reduce SSB consumption among youth (5). Policies that restrict the sale of SSBs in schools are one way to reduce children’s SSB consumption (6). However, emerging evidence suggests that these policies alone may not be sufficient to curb students’ SSB intake (7,8), in part because SSBs are often purchased at stores located near schools (9,10). Previous research suggests that as many as 65% of youth shop at such stores, where they frequently purchase high-calorie, low-nutrient foods and beverages (11). To date, only one study has examined how commonly students purchase foods and beverages while traveling to and from school (12), and no study has examined whether out-of-school beverage-purchasing behaviors are associated with in-school beverage consumption. Understanding these relationships is particularly important in low-income and minority communities, which tend to have a high concentration of stores that sell unhealthful foods and beverages (13,14).

The objective of this study was to describe where students from low-income, ethnically diverse communities obtain the SSBs they drink during school lunchtime and to examine whether students who purchase beverages while traveling to and from school are more likely to drink SSBs during school lunchtime. We hypothesized that students who purchase beverages on their school commute would be more likely to drink SSBs during school lunchtime, even in schools with limited access to SSBs from school sources. Findings can help advance policies to reduce SSB consumption in youth.

## Methods

This cross-sectional study used survey data collected from both intervention and control group students during the follow-up period of a larger school-based intervention promoting water access and intake. Details of the intervention, which took place in 12 middle schools in the San Francisco Bay Area from February through June 2013, are available elsewhere (15). Students in study schools were predominantly low-income and minority; more than 50% of students in study schools were eligible for free and reduced-price meals through the National School Lunch Program (a proxy for low household income) and were of Latino or African American race/ethnicity. Under California state law (16), study schools were not allowed to sell SSBs other than electrolyte replacement beverages (ie, sports drinks and flavored waters with electrolytes) and flavored low-fat milk. This study reports on data collected following the water promotion intervention. All procedures were approved by the Committee on Human Research at the University of California, San Francisco and by school district research committees when applicable.

At each school, a random sample of 60 students (stratified by grade level) in grades 6 through 8 who spoke either English or Spanish was selected to participate in study surveys. No other exclusion criteria were applied. We obtained passive (opt-out) parental consent. Parents received study information sheets (sent home via children). If parents did not want their child to participate, they could opt-out by signing a form and sending it to the teacher or by calling the research team directly.

Trained research staff administered surveys in a quiet location within 2 hours after students ate lunch. Surveys had approximately 60 items and took students 10 to 15 minutes to complete. We obtained written assent from students before data collection. Students answered questions about what beverages they consumed during school lunchtime, whether and where they purchase beverages on the way to and from school, and their sociodemographic characteristics. We held makeup sessions within 3 weeks after the initial survey date for students who were absent during the first survey administration. Data collection occurred in May and June 2013. Students received $5 gift cards for completing surveys.

A total of 720 students (60 from each of 12 schools) were randomly selected to participate in surveys. Of these, 605 (84.0%) students completed the follow-up surveys. We excluded data from 4 students (0.7% of the students who completed the follow-up surveys) in our analyses, because consistency and validity checks elicited concerns with the accuracy of the data. We also excluded students who had missing data on key study variables (n = 4; 0.7% of completed sample). Our final analytic sample was 597 students.

The sample was predominantly Latino (Table 1). About half (48.6%) of students were female, and approximately two-thirds (65.5%) spoke English at home. Most students (80.6%) were born in the United States.

**Table 1:** Sociodemographic Characteristics of Participants (N = 597) in Study of Sugar-Sweetened Beverages Among Middle School Students, San Francisco Bay Area, 2013

Characteristic	Value^a^
**Age, y, mean (SD)**	12.8 (0.96)
**Sex**	
Female	290 (48.6)
Male	307 (51.4)
**Grade**	
6th	152 (25.5)
7th	217 (36.3)
8th	228 (38.2)
**Language spoken at home**	
English	391 (65.5)
Other	206 (34.5)
**Race/ethnicity^b^**	
Latino	347 (58.1)
Black	129 (21.6)
Asian	100 (16.8)
White	90 (15.1)
Other	16 (2.7)
**Born in the United States**	
Yes	481 (80.6)

Abbreviation: SD, standard deviation.

a Values reported as n (%), unless otherwise indicated. Values may not sum to 100% because of missing data.

b Participants could select more than one race/ethnicity category; (n) % reflects number of students who marked yes in each category.

Drinking only a few sips of SSBs may not produce a clinically significant outcome. Thus, we repeated our analysis using a newly created outcome variable that coded students as drinking an SSB if they drank in total more than a few sips of SSBs (results not shown). We summed across the different types of SSBs to calculate the total amount of SSBs each student consumed and used this total to classify students in a dichotomous manner (yes/no) based on whether they drank in total more than a few sips of SSBs during school lunchtime. The pattern of results was identical across the 2 sets of analyses.

### Key measures

Our main outcome variable was students’ self-reported consumption of SSBs during school lunchtime. Students were asked whether they drank (yes/no) specified beverages during school lunchtime, including flavored waters, sports drinks, soda, energy drinks, and other sugary/sweetened drinks. If students marked that they drank a particular beverage, they were asked to 1) describe the beverage (brand, flavor, name), 2) indicate where they obtained the beverage (check all that apply: got it free with lunch, bought it at school, got it from a friend at school, brought it from home, bought it on the way to school, or somewhere else), and 3) indicate the amount they consumed (a few sips, less than 1 glass or half a bottle, 1 glass or half bottle, 2 glasses or 1 bottle, more than 2 glasses or 1 bottle). Questions were developed based on a previously validated survey of elementary students’ lunchtime food and beverage intake (17). Students were classified as drinking an SSB during school lunchtime if they reported drinking “a few sips” or more of any of the following beverages: flavored bottled water, sports drinks, soda, energy drinks, or other sugary/sweetened beverages.

Our predictor variable was the purchase of beverages while traveling to and from school. Students were asked, “Where do you buy drinks when you are going to and from school?” Response options were “I do not buy any drinks when I am going to and from school,” “convenience/corner stores,” “grocery stores or supermarkets,” “fast-food restaurants,” and “some other location.” We dichotomized students into 2 groups: those who purchased beverages from one or more of these locations when going to and from school and those who did not.

Students were also asked about sociodemographic characteristics that may be associated with their beverage consumption patterns, including their age (18), sex (19), race/ethnicity (20), language spoken at home (21,22), and birthplace (22). Students indicated their age in years and their sex (male/female). Students marked all race/ethnicity categories that applied in response to the question, “How do you describe yourself?” Response options were white, black, African American, Latino(a)/Hispanic, Filipino, Pacific Islander, other Asian, American Indian or Alaska Native, or some other race or ethnicity. Each race/ethnicity variable was treated as a separate indicator variable; students were coded as yes for each category they marked and no for each category they did not mark. For analyses, we combined Filipino, Pacific Islander, and other Asian into a single category (Asian) and combined Native American or Alaska Native and some other race or ethnicity into a single category (other race/ethnicity). Students were also asked “What language do you speak at home most of the time?”’ and could select up to 2 options (if they spoke 2 languages equally often at home) from the following: English, Spanish, Tagalog, Vietnamese, and other. We dichotomized students into those who spoke any English at home and those who spoke no English at home. Students were also asked whether they were born in the United States (yes/no). Finally, we used school enrollment rosters to determine students’ grade level (6th, 7th, or 8th).

Research staff entered survey data into a Research Electronic Data Capture (REDCap) secure electronic database (23). Means, standard deviations, and proportions were estimated for sociodemographic characteristics and for predictor and outcome variables. Multivariate logistic regression, adjusting for clustering at the school level and controlling for students’ sociodemographic characteristics, was used to examine the association between students’ purchase of beverages while traveling to and from school and their consumption of SSBs during school lunchtime. As an exploratory analysis, we also used logistic regression with purchasing behavior as the dependent variable and demographic characteristics as the predictor variables to describe the types of students who were most likely to purchase beverages while traveling to and from school. All analyses were performed using Stata version 13.1 (StataCorp LP) (24).

## Results

Approximately one-fifth (n = 122; 20.4%) of students reported drinking any amount of SSBs during school lunchtime. Some students reported consuming more than one beverage. Among students who drank any SSBs during school lunchtime (n = 122), the most popular SSBs were other sugary/sweetened beverages (eg, lemonade, fruit drinks; 40.2% of students [n = 49] reported consuming), sports drinks (34.4%, n = 42), and soda (27.9%, n = 34). A smaller proportion of the students who consumed any SSB reported drinking flavored bottled water (13.9%, n = 17) or energy drinks (10.7%, n = 13).

Students obtained the SSBs they consumed during school lunchtime from various sources (Table 2). Most of the SSBs were brought from home (34.1%), obtained from a friend at school (23.2%), or purchased while traveling to or from school (22.6%). Only sports drinks and flavored bottled water were obtained from school sources. There was variation in the locations where students obtained different beverages. Flavored bottled waters were most often obtained during the school commute, sodas were most often brought from home, and energy drinks were most often obtained from a friend at school.

**Table 2:** Sources of Sugar-Sweetened Beverages Among Middle School Students, by Beverage Type, San Francisco Bay Area, 2013

Beverage	Brought From Home	From a Friend	Bought Traveling to/From School	Bought at School	Other/ Missing	Free With Lunch
Flavored water	4	4	5	3	1	0
Soda	17	8	7	0	2	0
Sports drink	9	9	8	12	2	2
Energy drink	3	5	4	0	1	0
Other	20	10	11	0	8	0
Total	**53**	**36**	**35**	**15**	**14**	**2**

About half (50.1%) of students in the sample reported that they purchase beverages from one or more locations while going to and from school. Among these students, almost three-quarters (71.8%) reported that they purchase beverages from convenience/corner stores, 41.5% from grocery stores, 15.9% from fast-food restaurants, and 9.6% from some other location (eg, mobile vendors near schools).

In a logistic regression examining the association between students’ SSB consumption and their self-reported beverage-purchasing behavior, we found that students who reported that they buy beverages on their way to or from school were significantly more likely to report drinking an SSB at lunchtime compared with students who reported that they do not buy beverages during their school commute (adjusted odds ratio [AOR], 3.32; 95% confidence interval [CI], 2.19–5.05, *P* < .001) (Table 3). Female students were less likely than male students to report that they drank an SSB during school lunchtime, although the difference was not significant at the *P* = .05 level (AOR, 0.79; 95% CI, 0.61–1.02, *P* = .07).

**Table 3:** Association Between Middle School Students’ Consumption of SSBs During School Lunchtime and Purchase of Beverages While Traveling to and From School, San Francisco Bay Area, 2013

Characteristic	No. Who Drank SSBs^a^, N (%)	AOR^b^ (95% CI)	*P* Value
**Purchase beverages while traveling to/from school**			
Yes	88 (29.3)	3.32 (2.19–5.05)	<.001
No	34 (11.5)	1 [Reference]	
**Age**	—	0.80 (0.57–1.14)	.22
**Sex**			
Female	59 (19.2)	0.79 (0.61–1.02)	.07
Male	63 (21.7)	1 [Reference]	
**Grade**			
6th	35 (23.0)	1 [Reference]	
7th	45 (20.7)	1.05 (0.69–1.57)	.84
8th	42 (18.4)	1.04 (0.56–1.94)	.91
**Race/ethnicity^c^**			
Latino/Hispanic	74 (21.3)	1.42 (0.61–3.28)	.41
Black	29 (22.5)	1.17 (0.51–2.68)	.72
Asian	19 (19.0)	1.35 (0.52–3.47)	.54
White	17 (18.9)	1 [Reference]	
Other	5 (31.3)	1.75 (0.85–3.64)	.13
**Language spoken at home**			
English	82 (21.0)	0.92 (0.56–1.51)	.74
Other	40 (19.4)	1 [Reference]	
**Born in the United States**			
Yes	106 (22.0)	1.64 (0.86–3.11)	.13
No	16 (13.8)	1 [Reference]	

Abbreviations: AOR, adjusted odds ratio; CI, confidence interval.

a Students were classified as having had an SSB if they consumed any amount of any SSB at lunchtime.

b Logistic regression analysis adjusted for clustering at the school level.

c Values show the adjusted likelihood of self-identifying as that race/ethnicity vs not self-identifying as that race/ethnicity.

In logistic regression analyses with purchasing behavior as the dependent variable and student sociodemographic characteristics as predictor variables, we found that Asian students were less likely than white students to report buying beverages while traveling to and from school (AOR, 0.56; 95% CI, 0.36–0.87, *P* = .01). No other sociodemographic characteristics were associated with students’ beverage-purchasing behavior (Table 4).

**Table 4:** Association Between Demographic Characteristics and Middle School Students’ Purchase of Beverages While Traveling to and From School, San Francisco Bay Area, 2013

Characteristic	No. (%) Who Purchased Beverages	AOR^a^ (95% CI)	*P* Value
**Age**	—	0.90 (0.63–1.30)	.59
**Sex**			
Female	156 (50.6)	1.04 (0.74–1.46)	.81
Male	144 (49.4)	1 [Reference]	
**Grade**			
6th	68 (44.7)	1 [Reference]	
7th	108 (49.3)	1.33 (0.71–2.47)	.37
8th	124 (54.4)	1.89 (0.77–4.59)	.16
**Race/ethnicity^b^**			
Latino/Hispanic	170 (49.0)	1.05 (0.59–1.86)	.87
Black	80 (62.0)	1.57 (0.88–2.82)	.13
Asian	39 (39.0)	0.56 (0.36–0.87)	.01
White	47 (52.2)	1.06 (0.62–1.82)	.84
Other	10 (62.5)	1.32 (0.62–2.84)	.47
**Language spoken at home**			
English	211 (54.0)	1.40 (0.83–2.36)	.21
Other	89 (43.2)	1 [Reference]	
**Born in the United States**			
Yes	250 (52.0)	1.12 (0.73–1.71)	.60
No	50 (43.1)	1 [Reference]	

Abbreviations: —, not available; AOR, adjusted odds ratio; CI, confidence interval.

a Logistic regression analysis adjusted for clustering at the school level.

b Values show the adjusted likelihood of self-identifying as that race/ethnicity vs not self-identifying as that race/ethnicity.

## Discussion

In this cross-sectional study, we examined the prevalence of SSB consumption during school lunchtime among an ethnically diverse sample of students attending low-income middle schools in the San Francisco Bay Area. Several findings are noteworthy. First, despite having limited access to SSBs in school, more than 1 in 5 students in our sample reported drinking an SSB during school lunchtime. This finding is concerning, given that SSB consumption contributes to obesity, diabetes, and other chronic diseases (25).

In our sample, the only SSBs students obtained from school sources were sports drinks and flavored water. This finding is consistent with California Senate Bill 965, which limits the SSBs available in schools to electrolyte replacement beverages (16). Instead, most SSBs consumed during school lunchtime were obtained from nonschool sources, including from home (34.4%), from a friend (23.4%), and from stores or restaurants while traveling to or from school (22.7%).

After the passage of the Healthy, Hunger-Free Kids Act of 2010, the United States Department of Agriculture updated federal policy on the types of beverages schools participating in the National School Lunch Program are allowed to sell to students during the school day (26). The new regulations, which are more stringent than California’s Senate Bill 965, prohibit schools from offering nearly all calorically sweetened beverages. Flavored milk is allowed, and high schools (but not middle and elementary schools) can sell zero- or low-calorie beverages (≤40 calories per 8 ounces or ≤60 calories per 12 ounces). Although these regulations are an important step toward ensuring healthy school environments, our findings add to the growing evidence that policies need to also target nonschool environments to meaningfully influence students’ beverage intake (27).

Half of the students in our study reported they generally purchase beverages on their school commute. Corner stores and convenience stores were the most common location for making such purchases. These findings are similar to results reported by Vander Veur et al, who found that about 58% of fourth- through sixth-grade students in Philadelphia schools purchased food or beverages from corner stores on their way to or from school (12). Others have found that young people who shop at corner and convenience stores typically purchase SSBs rather than healthier beverages such as water or milk (11). Consistent with those findings, we found that students who purchased beverages while traveling to and from school were more than 3 times as likely to drink an SSB during school lunchtime as students who did not.

Interventions to improve youth’s beverage consumption may benefit from focusing on the home environment and on vendors frequented by students. For example, previous interventions have successfully worked with vendors to offer healthier options by providing storeowner training, cash incentives, and other support (28). Researchers have also found that posting signs in corner stores with beverage caloric information (particularly when displayed as physical activity equivalents) can reduce adolescents’ SSB purchases (29). Such interventions may be particularly important in low-income and racial/ethnic minority communities such as those included in this study, which tend to have higher concentrations of convenience stores and fast-food outlets than higher-income or predominantly white neighborhoods (13,14). Interventions might also take a programmatic or educational approach, for example by seeking to change norms around beverage consumption through peer-to-peer education (30).

We found that Asian students were less likely to purchase beverages than were white students during their school commute, and that female students were less likely than male students to consume SSBs during school lunchtime. Future studies could elucidate why these characteristics may be protective; public health professionals can then leverage those insights to develop tailored interventions.

Our study findings are subject to several limitations. We used cross-sectional data, so we cannot establish any causal relationships. We surveyed middle school students from primarily minority racial/ethnic backgrounds. Beverage consumption patterns tend to vary by race/ethnicity and age (18), so our results may not generalize to populations with different demographic characteristics. Study schools were located in urban environments in the San Francisco Bay area; our findings may not generalize to schools in more suburban or rural areas, particularly if students living in these areas encounter fewer or different types of retailers during their school commutes. Although we had a high response rate (over 85%), we did not collect data on nonrespondents, and our findings may not generalize to those students who did not complete surveys. We also did not collect data on students’ mode of transit to school (eg, walk, bicycle, car, bus). Students who actively commute (eg, walk or bike) may have more opportunities to purchase beverages during the school commute than students who are driven or take the bus. Additionally, about half of the students in our sample participated in a water promotion intervention before data collection for this study. Although the intervention had no effect on students’ SSB intake (results reported elsewhere) (15), the intervention may have affected these students’ survey responses in other ways. Finally, despite our efforts to check the consistency and accuracy of students’ survey responses, students may have misreported their beverage intake.

We found that SSB consumption is prevalent even in schools with limited access to SSBs from school sources. These findings suggest that interventions and policies to reduce SSB consumption may be more effective if they simultaneously address the environments both in and outside of schools. Future research is needed to assess what strategies are most effective at reducing youth SSB consumption. 
